# Prevalence of Hepatitis B and Hepatitis C Viral Infections and Their Associated Factors among Diabetic Patients Visiting Debre Tabor Referral Hospital, Northwest Ethiopia, 2021: A Cross-Sectional Study

**DOI:** 10.1155/2023/5077706

**Published:** 2023-11-17

**Authors:** Debaka Belete, Dessie Kassaw, Tesfaye Andualem

**Affiliations:** ^1^University of Gondar, College of Medicine and Health Sciences, School of Biomedical and Laboratory Sciences, Department of Medical Microbiology, Gondar, Ethiopia; ^2^Debre Tabor University, College of Health Sciences and School of Medicine, Department of Medical Laboratory, Debre Tabor, Ethiopia

## Abstract

**Background:**

Viral hepatitis is a global public health problem that affects millions of people each year, causing disability and death. Hepatitis B and C viruses are the most common causes of viral hepatitis and are associated with chronic liver disease, cirrhosis, and hepatocellular carcinoma. The primary site of infection for these viruses is the liver, the primary site of hormone and glucose metabolism closely linked to diabetes mellitus (DM), which is associated with increased morbidity and mortality worldwide. As a result, assessing the coexistence of viral hepatitis and DM could be important in disease management, prevention, and control measures in DM patients.

**Objective:**

The aim of our study is to assess the prevalence and associated factors of HBV and HCV among diabetes patients attending Debre Tabor Referral Hospital.

**Methods:**

An institutional-based, cross-sectional study was conducted from December 1, 2021, to February 30, 2021. A systematic sampling technique was used for selecting study participants. Serum samples were screened with a rapid test kit for hepatitis B (HBV) and hepatitis C (HCV) infections. A pretested structured questionnaire was constructed to collect the data, which were later analyzed using SPSS version 23. Inferential statistics were used to evaluate the associated risk factors for the outcome variable. A *p* value of <0.05 was considered statistically significant.

**Result:**

A total of 152 diabetes patients were included in this study, with 78 (51.3%) males and 74 (48.7%) females, with a mean age of 39.24 ± 17.90 years. The prevalence of HBV and HCV was 6 (3.9%) and 2 (1.3%), respectively. Most of potential risk factors such as, histories of surgical procedures, tooth extraction, hepatitis infection in the family, blood transfusion, alcohol consumption, body tattooing, and multiple sexual partners were not statistically significant for HBV and HCV infections.

**Conclusion:**

In this study, no association was obtained between sociodemographic, clinical, and behavioural factors and the prevalence of hepatitis B and C viruses. Furthermore, there is no significant association detected between HBV or potential HCV infection and DM. Despite these results, continuing professional training programs on HBV and HCV infection, including increased vaccination coverage rates for HBV, are required.

## 1. Introduction

Diabetes mellitus (DM) is a major global public health problem with a rapidly increasing incidence and prevalence, particularly in developing countries [1]. It causes a disease of chronic complications in diabetic patients that is characterised by chronic hyperglycemia and disturbances in carbohydrates, fat, and protein and is also secondary to defects in insulin secretion, action, or both metabolisms. Based on the pathogenic process, there are two types of diabetes mellitus: type 1 and type 2. Type 1 diabetes is the result of a complete or near-total insulin deficiency [2]. Type 2 DM is a heterogeneous group of disorders characterised by variable degrees of insulin resistance, impaired insulin secretion, and increased glucose production [3]. At least initially and frequently throughout their lives, these people do not require insulin treatment to survive; ketoacidosis rarely occurs spontaneously in this type of diabetes; when it does occur, it is usually associated with the stress of another illness, such as infection [4].

DM patients are highly susceptible to infectious diseases such as bacterial, fungal, parasitic, and viral diseases because of cellular immunity disorders and phagocyte dysfunction caused by hyperglycemia and decreased vascularization among viruses such as hepatitis B and C, which are the most common [5].

Viral hepatitis is a systemic disease that causes inflammation of the liver. Most cases of viral hepatitis in children and adults are caused mainly by viral hepatitis B and C [6]. Hepatitis B and C virus infections are major global health problems. According to estimations, 71 million people worldwide carry HCV chronically, while 257 million people worldwide have HBV chronic infection [7, 8].

The liver is the principal site of hormone and glucose metabolism, and about 30% of patients with cirrhosis have diabetes mellitus type 2 [9].

Some workers think that diabetes may be the cause of coexisting liver disease because cytoplasmic glycogen deposits, fat accumulation in hepatocytes, and presinusoidal fibrosis are seen both in diabetes and cirrhosis. Recently, diabetes has been implicated in the pathogenesis of cirrhosis through lesions of nonalcoholic steatohepatitis (NASH), and the progression of NASH to cirrhosis in diabetics has been reported [10].

Some studies show that DM2 patients have a higher risk of being infected with parentally transmitted viruses such as hepatitis B or C viruses since they undergo frequent hospitalization and are submitted to blood tests such as blood glucose monitoring [11]. Although 80–85% of individuals with hepatitis C virus (HCV) infection become chronic carriers at risk of developing cirrhosis and hepatocellular cancer, only around 90% of those with hepatitis B virus (HBV) infection progress spontaneously towards healing [12]. When a diabetic person contracts the infection, the risk is very high and the infection spreads more rapidly [13]. Chronic hepatitis C virus (HCV) infection itself also increases the risk of HCC. It leads to chronic inflammation of the liver and liver fibrosis, which may eventually progress to cirrhosis. For patients with hepatitis C cirrhosis, the risk of developing HCC is 0.54 to 2.0% per year [14, 15].

Conversely, other studies have shown that hepatitis might contribute to the development of diabetes [16, 17]. There is no study, particularly on hepatitis B and hepatitis C viral infections and associated factors, particularly among patients with diabetes visiting Debre Tabor Referral Hospital. It is known that hepatitis B and hepatitis C viral infections related to DM are dynamic and changing phenomena, and research on this event is needed in the healthcare setting. Therefore, our study tried to assess the coexistence of hepatitis B and hepatitis C Viral Infections and their associated factors among patients with diabetes visiting Debre Tabor Referral Hospital, northwest Ethiopia.

## 2. Methods and Materials

### 2.1. Study Design, Setting, and Period

An institutional cross-sectional study design was conducted at Debre Tabor Referral Hospital from December 1, 2021, to February 30, 2021. The hospital is a referral-level hospital that serves over three million inhabitants and residents of Debre Tabor. Debre Tabor city is located in the Gondar administrative zone of the Amhara National Regional State, 666 km north of the capital city, Addis Ababa. The city has one government hospital, three public health centres, four public health posts, and three private clinics.

### 2.2. Study Population, Sample Size, and Sampling Technique

Patients with diabetes mellitus scheduled for follow-up visits at Debre Tabor General Referral Hospital between December 1, 2021, and February 30, 2021. The sample size was calculated using a single population proportion formula as follows: *N* = *z*^2^*p* (1−*p*)/*w*^2^, where *N* = the number of study participants to be included in this study; *Z* = the standard normal distribution value at 95% CI, which is 1.96; and *P* = the previous study's HCV prevalence at Jimma, which was 9.9% [18]; and *W* = the margin of error, taken as 5%. Accordingly, the sample size was *N* = 138. By considering a 10% nonresponse rate, the required sample size will be 152. A systematic sampling technique was used to select study subjects among DM patients who had follow-up at Debre Tabor General Referral Hospital from December 1, 2021, to February 30, 2021.

### 2.3. Data Collection Tool, Procedure, and Data Quality Assurance

The data were collected using a structured questionnaire. The questionnaire contained consent, sociodemographic variables, and potential risk factors for HBV and HCV infection, which were developed by adapting different peer-reviewed literature studies. To ensure data quality, 5% of the questionnaire was pretested before the actual data collection process. In addition to this, the semistructured questionnaire was prepared in the English version, translated into the local language (the Amharic version), and then transcribed back to English to maintain its consistency. Moreover, adequate training was given to data collectors and supervisors. A senior laboratory technician collected 3 mL of venous blood in a sterile disposable vacationer tube, allowed it to clot for 20 minutes, and then centrifuged it at 3000 RPM for 5 minutes at room temperature to separate the serum from blood.

The serum sample was used for anti-HCV antibody screening and serologic status of HBsAg according to the manufacturer's instructions and strictly followed standard operational procedures during sample collection and laboratory investigation in order to maintain the quality of the study. Known positive and negative samples for HBV and HCV were used as the quality control. SPSS version 23 statistical software was used to double-check, enter, and analyze the data.

### 2.4. Data Analysis and Interpretation

The collected data were checked for completeness, and then, the data were entered into SPSS version 23. To summarise the data, a statistical analysis for descriptive statistics of the variables was computed. For categorical variables, frequencies and percentages were computed, while for continuous variables, the mean and standard deviation were calculated. The odds ratios (OR) with 95% confidence intervals (95% CIs) were calculated. All variables with a *p* value <0.25 (to control the effect of confounding) in the bivariate analysis were included in the multivariate logistic regression. In all cases, a *p* value of 0.05 was taken as a statistically significant association. Finally, the findings were represented with texts and tables. Finally, the findings were represented with texts and tables.

### 2.5. Ethical Consideration

The study was approved by the Debre Tabor University, College of Health Sciences, and Department of Medical Laboratory Science, Research, and Ethical Review Committee (permission letter's reference number: CHS/221/2013 in the Ethiopian calendar, Date 5/2/2013 E.C.). All eligible study participants were informed about the purpose of the study; however, the participants were given the full right to withdraw at any time from participating in the research process. All hepatitis B and C positive laboratory results were only available to the clinician who attended to the patient, and based on the results, the patient was treated accordingly.

## 3. Result

### 3.1. Sociodemographic Characteristics

During the study period, a total of 152 diabetic patients were included in the study. Of those, 78 (51.3%) were female and 74 (48.7%) were male. Within the mean age of 39.24 ± 17 years, the majority of study participants were under the age of 29.54 (35.5%), followed by 30- to 49-year-olds at 46 (30.3%) and 52 (34.2%) over 50 years. The majority of study participants (86/56.6%) were married, followed by singles (50/32.9%) and divorced (16.5%). Of the total study participants, 66 (43.4%) were unable to read and write, followed by 44 (28.9%) in primary school, and only 30 (19.7%) had a college diploma or higher. The majority of the participants were farmers 44 (28.9%), followed by governmental employers 32 (21.1%), and 25 (16.4%) were housewives. Among the 152 participants, 84 (55.3%) lived in urban areas, while 68 (44.7%) lived in rural areas (Table 1).

In this study, only two participants were vaccinated against HBV. Of the total, 38 (25%) of the participants in the study had tattoos, 61 (40.1%) drank alcohol, 32 (21.1%) had multiple sex partners, and 21 (13.8%) had tooth extractions. Of the total study participants, 12 (7.9%) had a history of blood transfusions, 11 (7.2%) had a history of surgery, and 21 (13.8%) had a family history of hepatitis infection (Table 2).

### 3.2. Seroprevalence of Hepatitis B and C Viruses

Out of 152 study participants, the overall seroprevalence of HBV and HCV infection among DM patients was 1.3% and 3.9%, respectively, of whom 5 (14.43%) were male and 3 (6.89%) were female. The seroprevalence of hepatitis infections in diabetics living in urban and rural areas was found to be 7 (4.6%) and 1 (0.7%), respectively. In terms of age groups, the highest prevalence of hepatitis infection was found in the age group of 29 years (1.3%), followed by the age groups of 30 to 49 years at 0.66% and those 50 years and older at 3.3%. Regarding HBV infection in terms of marital status, 2 (1.3%) of the diabetic patients who tested positive for HBV were married. In addition, the highest prevalence of HBsAg infection was found in urban residences (1.3%). None of the type 1 diabetics tested positive for HBV.

The burden of seropositivity against anti-HCV antibodies in diabetics was found to be 6/152 (3.9%). In terms of DM patient types, type 2 diabetics had the highest prevalence of HCV infection (5 (3.3%)). College and above school diabetic patients had the highest seroprevalence of HCV-antibody (2%), followed by secondary school (0.66%), primary school (0.66%), and unable to read and write (0.66%), in that order. Regarding residence, the highest prevalence of HCV was found in urban residences (3.3%). A total of 5 (3.3%) diabetic patients had total coinfection (Tables 3 and 4).

### 3.3. Bivariate and Multivariate Analyses of HBV and HCV with Potential Risk Factors

In this study, possible risk factors were examined, such as age, sex, marital status, education level, and residence. A bivariate logistic regression model was used to examine the histories of surgical procedures, tooth extraction, hepatitis infection in the family, blood transfusion, alcohol consumption, body tattooing, and multiple sexual partners. Most of the expected risk factors, such as a history of blood transfusion, a history of tooth extraction, multiple sexual partners, a history of surgical procedures, and body tattooing, were found to be statistically insignificant (*p* value >0.2) for HBV infection. From the total participants, 61 (40.1%) had a history of an alcohol drinking habit, while 38 (25% of them) had a history of tattooing, 32 (21.1%) had a history of multiple sexual partners, and 21 (13.8%) had a history of tooth extraction (Tables 5 and 6).

## 4. Discussion

Viral hepatitis B and C infections are a major public health issue around the world. 96% of all viral hepatitis-related deaths are caused by hepatocyte-specific hepatitis B and C virus infections [19]. This study has revealed the seroprevalence of HBV and HCV infections and associated factors among diabetic patients at Debre Tabor Referral Hospital. Our study included 152 participants. The magnitude of HBV infection 2 (1.3%) in our work is lower than that in the studies conducted in Ghana (5.5%), Taiwan (13.5%), Turkey (3.8%), and Woldia (3.7%) [20–23], respectively. The differences might be due to differences in sample size, differences in geographical location, or failure to identify infected patients because of the serologic window incubation period.

The overall seroprevalence of the HCV infection in the present study was found to be 3.9%. The finding is in agreement with a study conducted in Turkey in which patients with type 1 and type 2 diabetes mellitus had a prevalence of anti-HCV infection of 3.2% [23] and in France (3.1%) [24]. Our result, however, is lower than those of studies conducted in India 5.7% [25], Pakistan 14.9% [26], Taiwan 6.8% [21], Jimma 9.9% [27], and Adigrat 5.5% [28].

On the other hand, a higher burden of HCV infection we found was higher than the national prevalence for the general population from 20 to 69 years old which was (1.6%) Sudan, (1.7%) Saudi Arabia, and (1.9%) in the United States [26–28].

The discrepancy might be due to the potential variability of the diagnostic test kit employed, geographic location, awareness of transmission methods of HCV, and exposure to risk factors.

In this study, bivariate and multivariate logistic regression analysis results show that there is no association between HBV infection and demographic variables such as sex, marital status, educational status, residence, and occupational status [21].

In our study, HBV and HCV were not associated with the history of invasive procedures such as tooth extraction, surgical procedures, tattooing, or a family history of liver disease. This study agrees with a study conducted in Taiwan [21]. Other studies conducted in Adigrat and Woldia were reported. HBV and HCV infections were associated with a history of invasive procedures such as tooth extraction and tattooing, as well as a history of liver disease [23, 29].

On the other hand, a history of blood transfusion and a history of alcohol consumption were not associated with HBV and HCV in this study, which agrees with studies conducted in Taiwan, Ghana, Jimma, and Woldia [20, 21, 23, 30], respectively. In addition, in this study, the history of multiple sexual partners was not associated with HBV and HCV infection. This finding was contradicted by a study conducted in India [25].

In summary, the overall seroprevalence of HBV and HCV infection in patients with DM was 1.3% and 3.9%, respectively. The prevalence of HBV and HCV infection was higher in type 2 DM patients than that in type 1 DM patients. So type 2 DM patients would require necessary preventive measures such as a vaccine against HBV and awareness of the mode of transmission of the HBV and HCV infections among patients with diabetes. HBV and HCV were not associated with sociodemographic characteristics, clinical characteristics, or behavioural variables in our study; more research is needed to investigate these issues thoroughly. We did not use additional confirmatory tests, especially for participants who were positive on the screeching test, due to resource constraints. Furthermore, this study's cross-sectional design precluded drawing a pathophysiological causal inference between DM and the risk of HBV and HCV. So multicenter studies are needed to establish the association, elucidate the reason for the association, and determine other aspects of the relationship using confirmatory tests.

## Figures and Tables

**Table 1:** Sociodemographic characteristics of study participants from December 1, 2021, to February 30, 2021, at Debre Tabor Referral Hospital (*N* = 152).

Variables	Frequency (*N*)	Percent (%)
Sex		
Male	78	51.3
Female	74	48.7
Age		
<29	54	35.5
30–49	46	30.3
>50	52	34.2
Residence		
Urban	84	55
Rural	68	45
Marital status		
Married	86	56.6
Single	50	32.9
Divorced	16	10.5
Educational status		
Unable to read and write	50	32.9
Primary school	44	28.9
Secondary school	28	18.4
Collage and above	30	19.7
Occupational status		
Governmental employee	32	21.1
Housewife	25	16.4
Merchant	17	11.2
Farmer	44	28.9
Other	33	21.7

**Table 2:** Determinant factors for HBV and HCV infection among DM patients at Debre Tabor Referral Hospital from December 1, 2021, to February 30, 2021 (*N* = 152).

Variables	Category	Frequency	Percentage
History of previous surgery	Yes	11	7.2
	No	141	92.8

History of tooth extract	Yes	21	13.8
	No	131	86.2

History of blood transfusion	Yes	12	7.9
	No	140	92.1

Having body tattoo	Yes	38	25
	No	114	75

Having multiple sex partners	Yes	32	21.1
	No	120	78.9

Having a family history of hepatitis infection	Yes	21	13.8
	No	131	86.2

History of blood testing for hepatitis B and C	Yes	18	11.8
	No	134	88.2

Having alcohol drinking habit	Yes	61	40.1
	No	91	59.9

Taking HBV vaccine	Yes	2	1.3
	No	150	98.7

**Table 3:** HBsAg and anti-HCV prevalence in different sociodemographic variables among DM patients at Debre Tabor Referral Hospital from December 1, 2021, to February 30, 2021 (*N* = 152).

Variables	Hepatitis B infections		Hepatitis C infection		Total
	Positive	Negative	Positive	Negative	
Sex					
Male	2	76	3	75	78
Female	0	74	3	71	74
Age					
<29	0	54	2	52	54
30–49	1	45	0	46	46
>50	1	51	4	48	52
Residence					
Urban	2	82	5	79	84
Rural	0	68	1	67	68
Marital status					
Married	2	84	4	82	86
Single	0	50	1	49	50
Divorced	0	16	1	15	16
Educational status					
Unable to read and write	0	50	1	49	50
Primary	1	26	1	26	27
Secondary	0	28	1	27	28
Collage and above	1	29	3	27	30
Occupational status					
Governmental employer	1	31	3	29	32
Housewife	0	25	1	24	25
Merchant	1	16	0	17	17
Farmer	0	44	0	44	44
Others	0	33	2	31	33
Type of DM					
Type 1 DM	0	80	1	79	80
Type 2 DM	2	70	5	67	72

**Table 4:** Seroprevalence of HBV and HCV among diabetic patients at Debre Tabor Referral Hospital from December 1, 2021, to February 30, 2021 (*N* = 152).

Variables	Status	Numbers (%)
HBsAg	Positive	2 (1.3)
	Negative	150 (98.7)

Anti-HCV antibody	Positive	6 (3.9)
	Negative	146 (96.1)

Coinfected	Positive	5 (3.3)
	Negative	147 (96.7)

Total	Positive	8 (5.3)
	Negative	144 (94.7)

**Table 5:** Bivariate and multivariate analyses of HBV with potential risk factor**s** among diabetic patients at Debre Tabor Referral Hospital from December 1, 2021, to February 30, 2021 (*N* = 152).

Variables	Frequency *N* (%)		HBV infection		COR (95% CI)	AOR (95% CI)	*p* value
			Pos	Neg			
Multiple sex partners	Yes	32 (21.1%)	1	31	0.34 (0.23, 63.1)	5.5 (0.17, 71.1)	0.32
	No	120 (78.9%)	1	119	1		

Tattoo	Yes	38 (25%)	1	37	0.43 (0.18, 50.0)	2.2 (0.11, 45.8)	0.59
	No	114 (75%)	1	113	1		

Tooth extraction	Yes	21 (13.8%)	1	20	0.2 (0.39, 108)	7.5 (0.36, 156)	0.18
	No	131 (86.2%)	1	130	1		

Drinking alcohol	Yes	61 (40.1%)	1	60	0.66 (0.4, 10.8)	2.3 (0.66, 82.3)	0.64
	No	91 (59.9%)	1	90	1		

**Table 6:** Bivariate and multivariate analyses of HCV with potential risk factors among diabetic patients at Debre Tabor Referral Hospital from December 1, 2021, to February 30, 2021 (*N* = 152).

Variables	Frequency *N* (%)		HCV infection		COR (95% CI)	AOR (95% CI)	*p* value
			Pos	Neg			
Blood transfusion	Yes	12 (7.9%)	1	11	0.40 (0.04, 3.80)	0.31 (0.001, 7.28)	0.47
	No	140 (92.1%)	5	135	1		

Surgery	Yes	11 (7.2%)	5	6	0.36 (0.39, 3.45)	0.59 (0.02, 12.40)	0.73
	No	141 (92.8%)	1	140	1		

Tattoo	Yes	38 (25%)	4	34	0.65 (0.11, 3.72)	1 (0.16–6.73)	0.96
	No	114 (75%)	2	112	1		

Tooth extraction	Yes	21 (13.8%)	4	17	0.29 (0.55, 1.74)	0.28 (0.43–1.93)	0.20
	No	131 (86.2%)	2	129	1		

Drinking alcohol	Yes	61 (40.1%)	4	57	0.32 (0.05, 1.80)	0.30 (0.04, 1.950)	0.20
	No	91 (59.9%)	2	89	1		

## Data Availability

The datasets used and/or analyzed during the current study are available within the article.

## Abbreviations

(Anti-HCV): Antibody against hepatitis C virus
(CI): Confidence interval
(CSA): Central Statistical Agency of Ethiopia
(DM): Diabetes mellitus
(DNA): Deoxyribonucleic acid
(G.C): Gregorian calendar
(HBV): Hepatitis B virus
(HBsAg): Hepatitis B surface antigen
(HCC): Hepatocellular carcinoma
(HCV): Hepatitis C virus
(IDU): Injection drug users
(NCDs): Noncommunicable diseases
(RNA): Ribonucleic acid
(RPR): Revolution per minute
(SPSS): Statistical Package for Social Sciences
(WHO): World Health Organization.

## References

[1] Danaei G., Finucane M. M., Lu Y. (2011). National, regional, and global trends in fasting plasma glucose and diabetes prevalence since 1980: systematic analysis of health examination surveys and epidemiological studies with 370 country-years and 2·7 million participants. *The Lancet*.

[2] World health organization (1999). *Definition, Diagnosis and Classification of Diabetes Mellitus and its Complications: Report of a WHO Consultation. Part 1, Diagnosis and Classification of Diabetes Mellitus*.

[3] Begic E., Arnautovic A., Masic A. (2016). Assessment of risk factors for diabetes mellitus type 2. *Materia Socio Medica*.

[4] Inzucchi S. E., Bergenstal R. M., Buse J. B. (2015). Management of hyperglycemia in type 2 diabetes, 2015: a patient-centered approach: update to a position statement of the American Diabetes Association and the European Association for the Study of Diabetes. *Diabetes Care*.

[5] Gutiérrez-Grobe Y., Ponciano-Rodríguez G., Méndez-Sánchez N. (2011). Viral hepatitis infection and insulin resistance: a review of the pathophysiological mechanisms. *Salud Pública de México*.

[6] Million Y., Teklu T., Alemu S., Ferede A., Belachew T., Desta K. (2019). Hepatitis B and hepatitis C viral infections and associated factors among patients with diabetes visiting gondar referral teaching hospital, Northwest Ethiopia: a comparative cross-sectional study. *Journal of Hepatocellular Carcinoma*.

[7] Liu J., Liang W., Jing W., Liu M. (2019). Countdown to 2030: eliminating hepatitis B disease, China. *Bulletin of the World Health Organization*.

[8] Lazarus J. V., Wiktor S., Colombo M., Thursz M. (2017). Micro-elimination–A path to global elimination of hepatitis C. *Journal of Hepatology*.

[9] Franco E., Bagnato B., Marino M. G., Meleleo C., Serino L., Zaratti L. (2012). Hepatitis B: epidemiology and prevention in developing countries. *World Journal of Hepatology*.

[10] Qureshi H., Ahsan T., Mujeeb S. (2002). Diabetes mellitus is equally frequent in chronic HCV and HBV infection. *Journal of Pakistan Medical Association*.

[11] Garcia-Compean D., Jaquez-Quintana J. O., Gonzalez-Gonzalez J. A., Maldonado-Garza H. (2009). Liver cirrhosis and diabetes: risk factors, pathophysiology, clinical implications and management. *World Journal of Gastroenterology*.

[12] Pichard E., Hépatites V., Gentilini M. (2012). *Éditeurs Médecine Tropicale 6ème Édition*.

[13] Seeff L. B. (2002). Natural history of chronic hepatitis C. *Hepatology*.

[14] Fattovich G., Pantalena M., Zagni I., Realdi G., Schalm S. W., Christensen E. (2002). Effect of hepatitis B and C virus infections on the natural history of compensated cirrhosis: a cohort study of 297 patients. *American Journal of Gastroenterology*.

[15] Fattovich G., Giustina G., Degos F. (1997). Effectiveness of interferon alfa on incidence of hepatocellular carcinoma and decompensation in cirrhosis type C. *Journal of Hepatology*.

[16] Naing C., Mak J. W., Ahmed S. I., Maung M. (2012). Relationship between hepatitis C virus infection and type 2 diabetes mellitus: meta-analysis. *World Journal of Gastroenterology*.

[17] Reilly M. L., Schillie S. F., Smith E. (2012). Increased risk of acute hepatitis B among adults with diagnosed diabetes mellitus. *Journal of Diabetes Science and Technology*.

[18] Qureshi S. A. (2007). Hepatitis C virus—biology, host evasion strategies, and promising new therapies on the horizon. *Medicinal Research Reviews*.

[19] Birku T., Gelaw B., Moges F., Assefa A. (2015). Prevalence of hepatitis B and C viruses infection among military personnel at bahir dar armed forces general hospital, Ethiopia. *BMC Research Notes*.

[20] Osakunor D., Adoba P., Sakyi S., Anto E., Ephraim R., Nsiah P. (2014). Seroprevalence of hepatitis B and C viral infections among type 2 diabetics: a cross-sectional study in the Cape Coast Metropolis. *Annals of Medical and Health Sciences Research*.

[21] Chen H.-F., Li C.-Y., Chen P., See T.-T., Lee H.-Y. (2006). Seroprevalence of hepatitis B and C in type 2 diabetic patients. *Journal of the Chinese Medical Association*.

[22] Gulcan A., Gulcan E., Toker A., Bulut İ, Akcan Y. (2008). Evaluation of risk factors and seroprevalence of hepatitis B and C in diabetic patients in Kutahya, Turkey. *Journal of Investigative Medicine*.

[23] Mekonnen D., Gebre-Selassie S., Fantaw S., Hunegnaw A., Mihret A. (2014). Prevalence of hepatitis B virus in patients with diabetes mellitus: a comparative cross sectional study at Woldiya General Hospital, Ethiopia. *The Pan African medical journal*.

[24] Rudoni S., Petit J., Bour J. (1999). HCV infection and diabetes mellitus: influence of the use of finger stick devices on nosocomial transmission. *Diabetes and Metabolism*.

[25] Juttada U., Smina T., Kumpatla S., Viswanathan V. (2019). Seroprevalence and risk factors associated with HBV and HCV infection among subjects with type 2 diabetes from South India. *Diabetes Research and Clinical Practice*.

[26] Madny A. G., Adam A. A. (2014). Seroprevalence of Hepatitis C Virus among type 2 diabetes mellitus patients in blue nile state, Sudan. *American Journal of Research Communication*.

[27] Esparza-Martín N., Hernández-Betancor A., Suria-González S. (2013). Serology for hepatitis B and C, HIV and syphilis in the initial evaluation of diabetes patients referred for an external nephrology consultation. *Nefrologia*.

[28] Sangiorgio L., Attardo T., Gangemi R., Rubino C., Barone M., Lunetta M. (2000). Increased frequency of HCV and HBV infection in type 2 diabetic patients. *Diabetes Research and Clinical Practice*.

[29] Gebremeskel T. K. (2017). *Sero Prevalence Of Hepatitis C Virus In Type Ii Diabetic Patients*.

[30] Ali S., Abera S., Mihret A., Abebe T. (2012). Association of hepatitis C virus infection with type II diabetes in Ethiopia: a hospital-based case-control study. *Interdisciplinary Perspectives on Infectious Diseases*.

